# Supplementary material for: Bap-Independent Biofilm Formation in Staphylococcus xylosus
Source: Microorganisms. 2021 Dec 17;9(12):2610. doi: 10.3390/microorganisms9122610 (PMC8708073; doi:10.3390/microorganisms9122610)
Supplement: Supplementary file 1 [file microorganisms-09-02610-s001.zip › microorganisms-1511819-supplementary.pdf]

Table S1: Identified peptide sequences (intensity values) mapping on Bap in wildtype and mutant strains of TWW strains 2.1023 and 2.1523

identified peptide sequences mapping on Bap	start	end	WT_.023 Intensity	Mut_.023 Intensity	WT_.523 Intensity	Mut_.523 Intensity
AATDDNDKTEEAESQLETELATSK	254	277	437230000		825520000	
AEPNSSVTVGFPGGGK	970	985			32081000	
AMATPTALAAAADQSEEVEK	347	366	208800000		327180000	
ASLIELVNDQESAK	322	335			629790000	
DAEYDDQGNLIR	686	697	168230000		437700000	
DEETTSESLQPSSEEDNTK	231	249			11131000	
DKGEALDVSDIK	58	69	442310000		834900000	
DLLPYIESVELHK	604	616			39975000	
DLLPYIESVELHKYDYQGLSGFDK	604	627			51671000	
ESDDTNKEDLMSPSDK	137	152	51506000		95708000	
GLLINNTLGTSTLALQDYDKDGLLDR	717	742			390300000	
GLLINNTLGTSTLALQDYDKDGLLDRYER	717	745	592080000		655690000	
IELDDTVANILNNAGSLENNK	486	506	51193000		146510000	
IELDDTVANILNNAGSLENNKLNYQMFVR	486	514	177740000		218030000	
LDLILAEHVTK	420	430	280790000		363530000	
LIEFNNLLPETIGVR	656	670	310470000			
LNQSVNNILTK	675	685	137520000	75044000	269420000	
LTNTWEVNFIR	454	464	196220000		340010000	
NETPSTEDKETTNSEEVLNENTDSEK	180	205			51684000	
NGVFSYSK	582	589	76472000		112630000	
QKEDFTFAGYLTDSK	702	716	171600000		181760000	
QVSLSDPENNDTDGDGKNDGDEVINYK	746	772	242550000		377590000	
SDEPKDEETTSESLQPSSEEDNTK	226	249	97864000		173990000	
SDEPKDEETTSESLQPSSEEDNTKETEK	226	253	242560000		265260000	
SFLSDQLPEK	285	294	211740000			
SNQWSYNCQIDK	592	603			31703000	
SNQWSYNCQIDKDLLPYIESVELHK	592	616			58891000	
SNQWSYNCQIDKDLLPYIESVELHKYDYQGLSGFDK	592	627	119000000		212380000	
SQPVEFVR	439	446	174800000			
SVEDSNADETNNEAK	70	84	219120000		318450000	
TLATPTR	336	342	12563000		26559000	
TSPLVGKPEAADITNEDTVVNGSVPLK	773	799	46138000		190040000	
TSPLVGKPEAADITNEDTVVNGSVPLKEGAASQAAK	773	808			596210000	
TSPLVGKPEAADITNEDTVVNGSVPLKEGAASQTAK	773	808	392440000			
TTAEDNSQKDNTQEDTATQK	206	225	394100000		544760000	
VADLTLDDIGNGSITSDNLNK	635	655	110330000		213300000	
VEDSNNSSNVVENNR	2097	2111	36659000		28438000	
YDYQGLSGFDK	617	627	96400000		180680000	

Supplementary figures

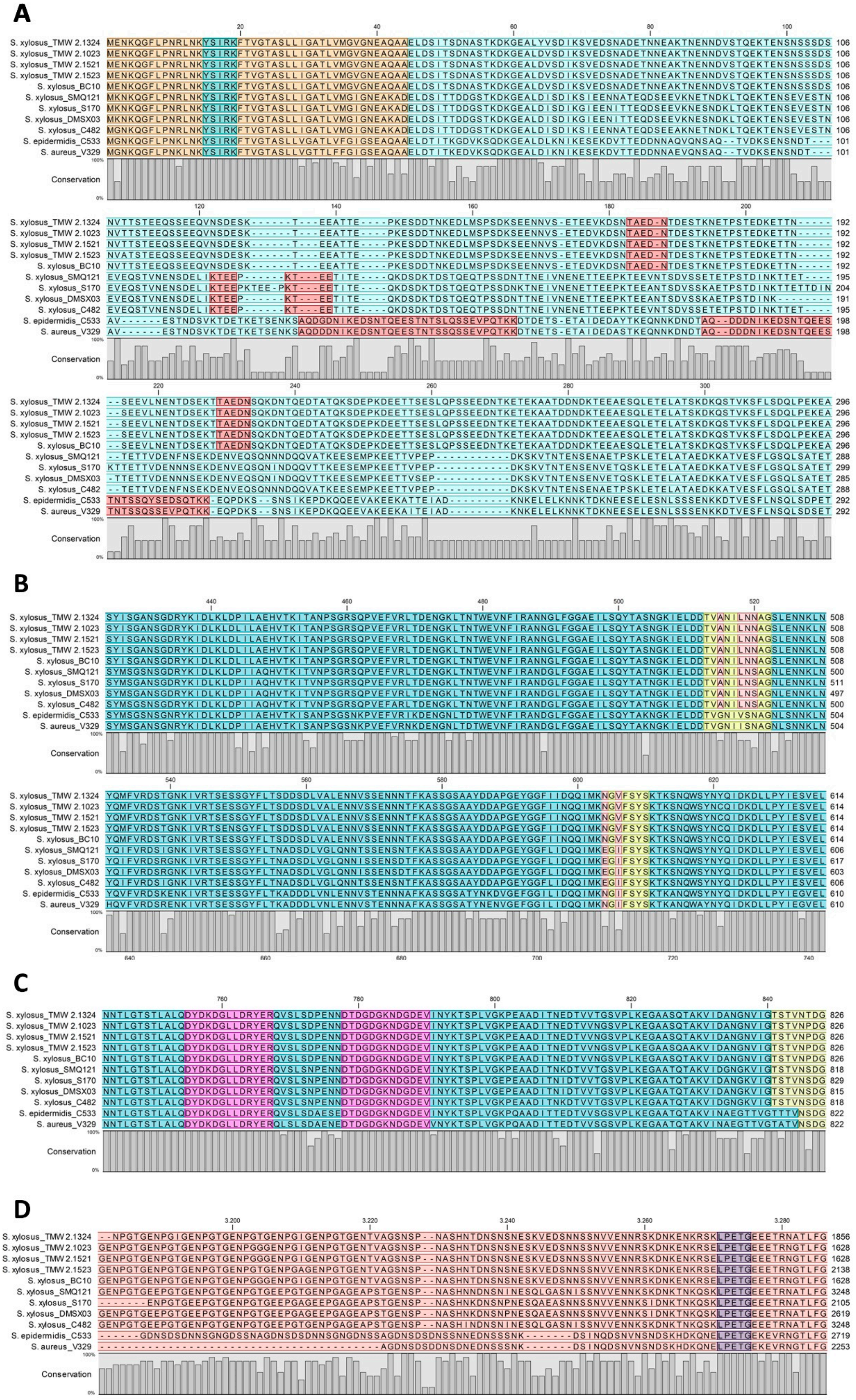
A
B
C
D
Conservation

Figure S1: Bap sequence alignment of selected *S. xylosus* strains with two Bap-dependent biofilm formers: *S. epidermidis* C533 and *S. aureus* V329. Only functionally important parts of the alignment are shown. A. shows the YSIRK-Signal peptide sequence as well the difference in A-region repeat length between *S. epidermidis*/*S. aureus* and *S. xylosus*. B. shows the sequence differences between the two amyloidprone peptides (defined by Taglialegna et al., [8]) of Bap Region B (marked in yellow). C. displays the conservation across species of EF hand domains 2 and 3 (pink). D. shows the differences in D-repeats between the species. While *S. aureus* and *S. epidermidis* region D is rich in SD repeats, *S. xylosus* encodes G-rich repeats.

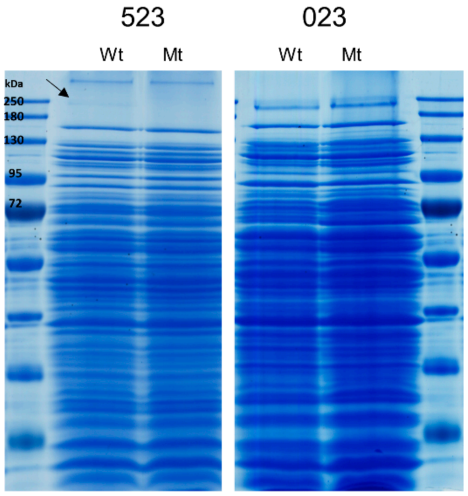

Figure S2: Analysis of cell extract protein preparations on SDS-PAGE. Left: TMW 2.1523 wildtype (Wt) and mutant (Mt) strain, Right: TMW 2.1023 Wt and Mt. The black arrow indicates a possible location of Bap in TMW 2.1523 - Wt.

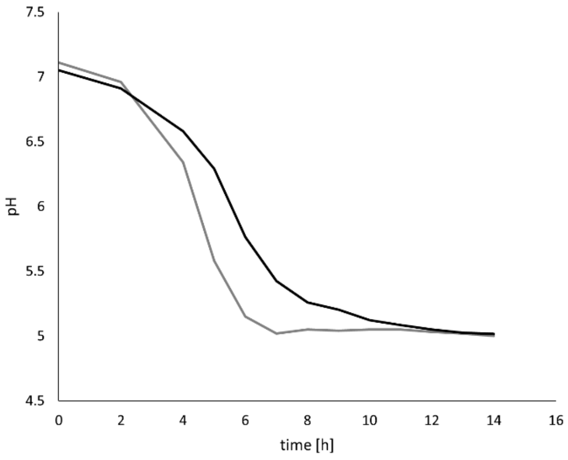

Figure S3: pH changes of S. xylosus TMW 2.1023 (grey) and 2.1523 (black) incubated in TSB^+ aerobically at 37 °C. Changes in pH were recorded over 12 hours, OD_{600} at t_0 was set to 0.1. Curves display the mean of 3 biological replicates.

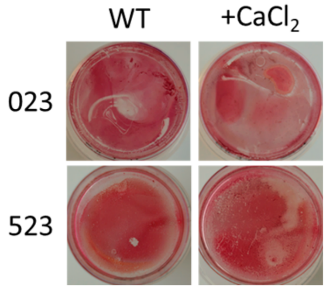

Figure S4: Calcium (20 mM) does not impair biofilm formation of selected *S. xylosus* strains (incubated in TSB$^+$, 24hrs, 37 °C, stained with safranin-O)

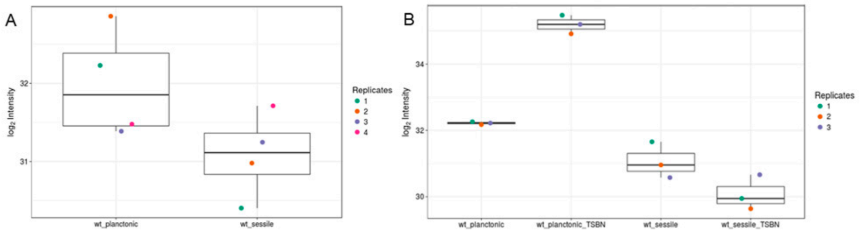

Figure S5: Boxplot of log_2 transformed LFQ-intensities measured for Bap in TMW 2.1023 (A) under planktonic and sessile conditions in Lac$^+$, for TMW 2.1523 (B) under planktonic and sessile conditions in Lac$^+$ and TSB_N.

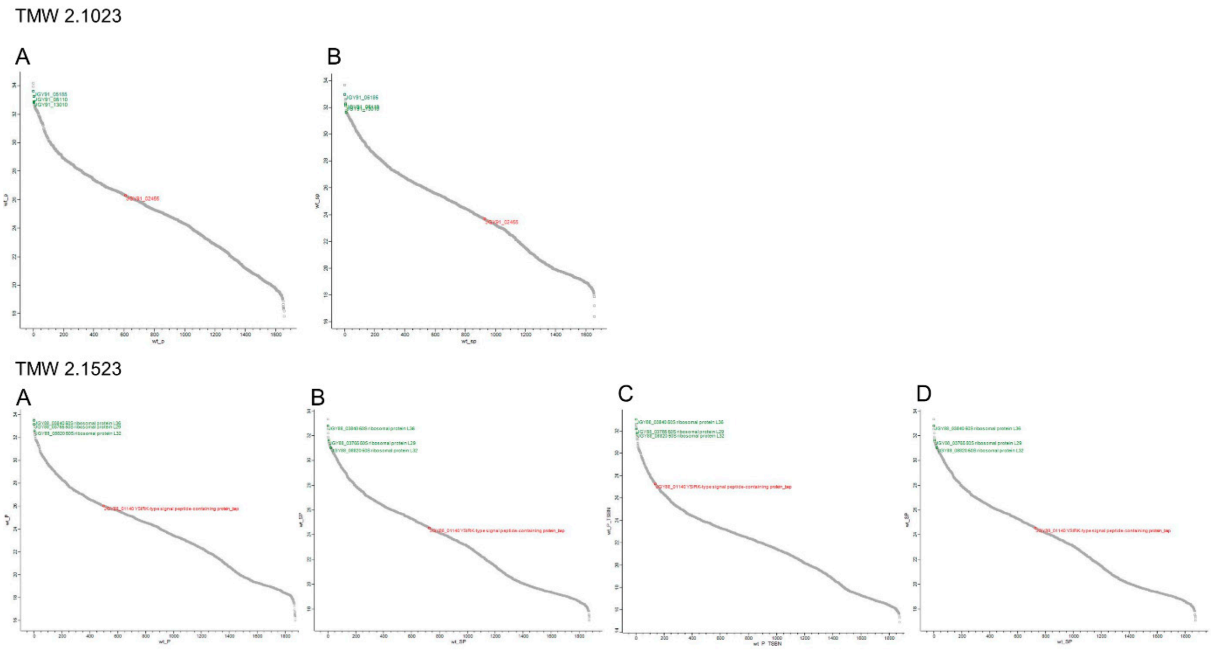

Figure S6: iBAQ-intensities of proteins expressed in TMW 2.1023 under planktonic (A) or sessile (B) conditions and TMW 2.1523 under planktonic Lac$^+$ (A), sessile Lac$^+$ (B), planktonic TSB_N (C) and sessile TSB_N (D) conditions. Bap is marked in red, highly abundant ribosomal proteins (50S) are marked in green for comparison.
